# The Its Not JUST Idiopathic pulmonary fibrosis Study (INJUSTIS): description of the protocol for a multicentre prospective observational cohort study identifying biomarkers of progressive fibrotic lung disease

**DOI:** 10.1136/bmjresp-2019-000439

**Published:** 2019-06-04

**Authors:** Fasihul Khan, Iain Stewart, Lucy Howard, Tricia M McKeever, Steve Jones, Glenn Hearson, Rebecca Braybrooke, Colin Edwards, Gisli Jenkins, Gauri Saini

**Affiliations:** 1 Respiratory Medicine, University of Nottingham, Clinical Sciences Building, Nottingham City Hospital, Hucknall Road, Nottingham, UK; 2 Division of Epidemiology and Public Health, University of Nottingham, Clinical Sciences Building, Nottingham City Hospital, Hucknall Road, Nottingham, UK; 3 Respiratory Medicine, University of Nottingham, Clinical Sciences Building, Nottingham City Hospital, Hucknall Road, Nottingham, UK; 4 Action for Pulmonary Fibrosis, City Wharf, Davidson Road, Lichfield, Staffordshire, UK; 5 Respiratory Medicine, University Of Nottingham, Nottingham, UK; 6 patientMpower Ltd, The Digital Depot, Thomas Street, Dublin, Ireland

**Keywords:** interstitial fibrosis, asbestos induced lung disease, hypersensitivity pneumonitis

## Abstract

**Introduction:**

The Its Not JUST Idiopathic pulmonary fibrosis Study (INJUSTIS) is a multicentre, prospective, observational cohort study. The aims of this study are to identify genetic, serum and other biomarkers that may identify specific molecular mechanisms, reflecting disease endotypes that are shared among patients with progressive pulmonary fibrosis regardless of aetiology. Furthermore, it is anticipated that these biomarkers will help predict fibrotic activity that may identify patterns of disease behaviour with greater accuracy than current clinical phenotyping.

**Methods and analysis:**

200 participants with the multidisciplinary team confirmed fibrotic lung disease (50 each of rheumatoid-interstitial lung disease (ILD), asbestosis, chronic hypersensitivity pneumonitis and unclassifiable ILD) and 50 idiopathic pulmonary fibrosis participants, recruited as positive controls, will be followed up for 2 years. Participants will have blood samples, lung function tests, quality of life questionnaires and a subgroup will be offered bronchoscopy. Participants will also be given the option of undertaking blinded home handheld spirometry for the first 3 months of the study. The primary end point will be identification of a biomarker that predicts disease progression, defined as 10% relative change in forced vital capacity (FVC) or death at 12 months.

**Ethics and dissemination:**

The trial has received ethical approval from the National Research Ethics Committee Nottingham (18/EM/0139). All participants must provide written informed consent. The trial will be overseen by the INJUSTIS steering group that will include a patient representative, and an independent chairperson. The results from this study will be submitted for publication in peer-reviewed journals and disseminated at regional and national conferences.

**Trial registration number:**

NCT03670576.

## Introduction

Interstitial lung diseases (ILDs) are a group of immunoinflammatory and fibrotic diseases of the lung parenchyma. In a substantial number of patients there is progressive fibrosis of the alveoli and interstitium that leads to increasing disability and ultimately the death of patients with these diseases. Establishing the aetiology of these fibrotic lung diseases is often a clinical challenge and the relevance of aetiology to disease behaviour remains controversial. The best characterised fibrotic ILD is idiopathic pulmonary fibrosis (IPF), which has a median survival of 3 years, and 5-year survival of 25%, which is worse than most cancers.^1^ Other conditions characterised by progressive pulmonary fibrosis include asbestosis, chronic hypersensitivity pneumonitis (HP), rheumatoid arthritis-associated ILD (RA-ILD), where the aetiology is assumed, and unclassifiable ILD where the clinical phenotype does not precisely reflect IPF.^2^ The progression of these related conditions is also remorseless, and their genetic predisposition similar to IPF, raising the possibility of shared targetable mechanisms across disease phenotypes regardless of likely aetiology.

Recently two drugs, pirfenidone^3 4^ and nintedanib,^5^ have been approved for the treatment of IPF. While these drugs are described as ‘anti-fibrotic’, they can only be prescribed for IPF, rather than all forms of progressive fibrotic disease.^6^ Therefore, patients with pulmonary fibrosis where the aetiology has been assumed, or the clinical features aren’t specific for IPF, cannot receive antifibrotic therapy at the current time. However, the risk factors and molecular pathways driving fibrosis in aetiologically defined or phenotypically unusual pulmonary fibrosis may be similar to IPF, thus potentially resulting in a large number of patients not having access to life-prolonging therapy. Our understanding of IPF has improved significantly both in terms of biomarkers^7 8^ and clinical end points.^9^ However, there remains a significant gap in our understanding of non-IPF ILD, with currently no approved treatments or cure.

RA-ILD, seen in 5%–10% of patients with rheumatoid arthritis remains a significant life-limiting complication with mortality in excess of 10% compared with patients without ILD. Subclinical interstitial lung abnormalities (ILAs) are seen in 30%–50% but individual risk of progression to ILD is unknown.^10^ Chronic HP diagnosis rests on history of antigen exposure and radiological appearance, which often has an overlap with other ILDs. Sometimes, there is no known antigen exposure, and more recent hypotheses suggest a combination of exposure in genetically predisposed individuals.^11 12^ While acute HP has a good prognosis, chronic HP is a progressive disease lacking evidence-based treatments with current therapy relying on immunosuppression. Despite improvements in radiology and the advent of multidisciplinary teams (MDTs), unclassifiable ILD remains a significant burden of ILD in clinical practice and represents between 10% and 38% of all ILDs.^13 14^ These patients present a diagnostic challenge and again no evidence-based treatments are available.

Recent studies have highlighted phenotypical and molecular similarities across a range of ILDs. For example, the minor allele frequency of the MUC5B promoter single-nucleotide polymorphism (SNP), widely associated with IPF,^15^ is found with increasing frequency in patients with chronic HP.^16^ Short telomeres have also been associated with RA-ILD^17^ and chronic HP, resulting in a prognosis similar to patients with IPF.^16^ Patients with RA-ILD and usual interstitial pneumonia pattern have radiological changes that are indistinguishable from IPF and the presence of traction change and honeycombing is associated with poor outcomes regardless of aetiology.

Together, these features suggest that there may be shared mechanisms in the progression of pulmonary fibrosis common among patients with lung fibrosis due to a number of aetiologies. To explore this hypothesis, the Its Not JUST Idiopathic pulmonary fibrosis Study (INJUSTIS) will recruit a clinical cohort comprising of ILD subgroups, to explore genetic, serum and clinical biomarkers that may distinguish progressive fibrosing lung disease phenotypes regardless of aetiology. This may then eventually enable therapeutics targeting specific mechanisms of disease rather than clinical phenotypes of disease.

## Methods and analysis

### Objectives

INJUSTIS is a prospective multicentre observational cohort study that will be managed through the Nottingham Respiratory Research Unit and funded by the National Institute of Health Research (NIHR) through the Nottingham Biomedical Research Centre and an NIHR professorship (RGJ).

The primary objective is to:

- Identify biomarkers that determine progressive fibrotic lung disease irrespective of aetiology.

The secondary objectives are to:

- Identify biomarkers that predict all-cause mortality.
- Identify biomarkers that predict changes in QoL scores.
- Identify biomarkers that predict the development of disease complications (respiratory failure and acute exacerbations).
- Investigate genetic association and epigenetic modifications which affect fibrotic disease severity and progression.
- Prospectively evaluate longitudinal disease behaviour in patients with non-IPF fibrotic lung diseases with a view to developing composite clinical end points for subsequent use in intervention studies.
- Explore association of environmental exposures with disease progression and all-cause mortality.
- Investigate whether home handheld spirometry over 3 months predicts disease progression and survival.

The primary end point will be:

- Disease progression defined as relative forced vital capacity decline ≥10% or death within 12 months.

Secondary end points are:

- All-cause mortality at time of censoring.
- Number of acute exacerbations over 2 years.
- Change in Quality of Life (QoL) Questionnaire Scores from baseline to 12 weeks.
- Rate of change in biomarker activity from baseline to 12 weeks.
- Change in diffusing capacity of the lung for carbon monoxide (DLco) from baseline to 12 months.
- Change in 6 min walk distance from baseline to 12 months.
- Change in transcriptomic profiles from baseline to 12 weeks.
- Change in home handheld spirometry values from baseline to 12 weeks.

### Selection of participants

Two hundred participants with recently diagnosed (within 18 months of study start date) fibrotic lung disease (50 each of rheumatoid-ILD, asbestosis, chronic HP and unclassifiable ILD) and 50 IPF participants as positive controls will be recruited from ILD clinics locally and across the UK. Only participants with MDT confirmed diagnosis of fibrotic ILD with radiological evidence of parenchymal lung fibrosis evidenced by reticulation and traction bronchiectasis, with or without honeycomb change will be recruited. Those with inflammatory radiological changes without evidence of fibrosis will not be deemed suitable regardless of clinical phenotype.

Recruitment will be reviewed on an ongoing basis and should rates fall below the expected levels, additional National Health Service (NHS) sites within the UK will be considered for participation. Eligible patients who meet the inclusion/exclusion criteria will be invited to consent. Most participants will be identified through outpatient clinics, but recruitment will not be restricted to this route. It will be explained to participants that entry into the study is entirely voluntary and that further treatment and care will not be affected by a decision to not partake. It will be clearly stated that participants are free to withdraw from the trial at any time. All participants will provide written informed consent, which will be countersigned by a member of the study team.

Inclusion criteria:

- Male or female aged ≥18 years.
- Able and willing to give written informed consent.
- Recently diagnosed (defined as diagnostic CT scan or surgical lung biopsy (if applicable) within 18 months of study start date).
- An MDT diagnosis of fibrotic ILD defined as the presence of traction change and reticulation with or without honeycombing within the lung parenchyma associated with either:
  - Rheumatoid arthritis (rheumatologist diagnosed with anti-cyclic citrullinated peptide antibodies and/or rheumatoid factor positive).
  - Asbestosis (appropriate occupational history and radiological evidence of asbestos exposure).
  - Chronic HP in accordance with consensus criteria^11^ (appropriate exposure history, radiological features ± avian and fungal precipitins).
  - Unclassifiable fibrotic lung disease (fibrotic lung disease otherwise unclassifiable despite extensive clinical and radiological examination).
  - IPF in accordance with consensus criteria (American Thoracic Society (ATS), European Respiratory Society (ERS), Japanese Respiratory Society (JRS, Latin American Thoracic Society (ALAT) guidelines).^18 19^

Exclusion criteria:

- Participating in an interventional clinic trial.
- Asymptomatic ILAs and normal lung function.
- Change in clinical phenotype from initial radiological diagnosis to screening.
- Any connective tissue disease other than rheumatoid arthritis.
- Acute HP.
- Participants who do not possess a smartphone cannot partake in the domiciliary spirometry.

#### Study regimen

Both cases and IPF controls will undertake the same investigations. Following informed consent, the following test results, previously carried out as part of the participant’s usual NHS care, will be used for the purposes of the study:

- HRCT findings.
- Blood results.
- Lung function tests.
- Bronchoscopy samples if already taken.

All participants will have baseline investigations at the first visit having provided informed consent. At the first visit, 40 mL of blood will be obtained, full lung function tests and a 6 min walk test will be performed. Participants will also be asked to complete QoL Questionnaires (Medical Research Council Dyspnoea Scale,^20^ Leicester Cough Questionnaire,^21^ IPF-abridged Profile for Assessment and Referral to Care,^22^ King’s Brief ILD Questionnaire^23^ and EQ-5D-5L).^24^ If consent is given for optional bronchoscopy, this will be subsequently performed and bronchoalveolar lavage carried out. Participants with a smartphone will be given the option of a home handheld spirometer and asked to provide daily FVC readings for the first 3 months of the study period.

**Figure 1:**
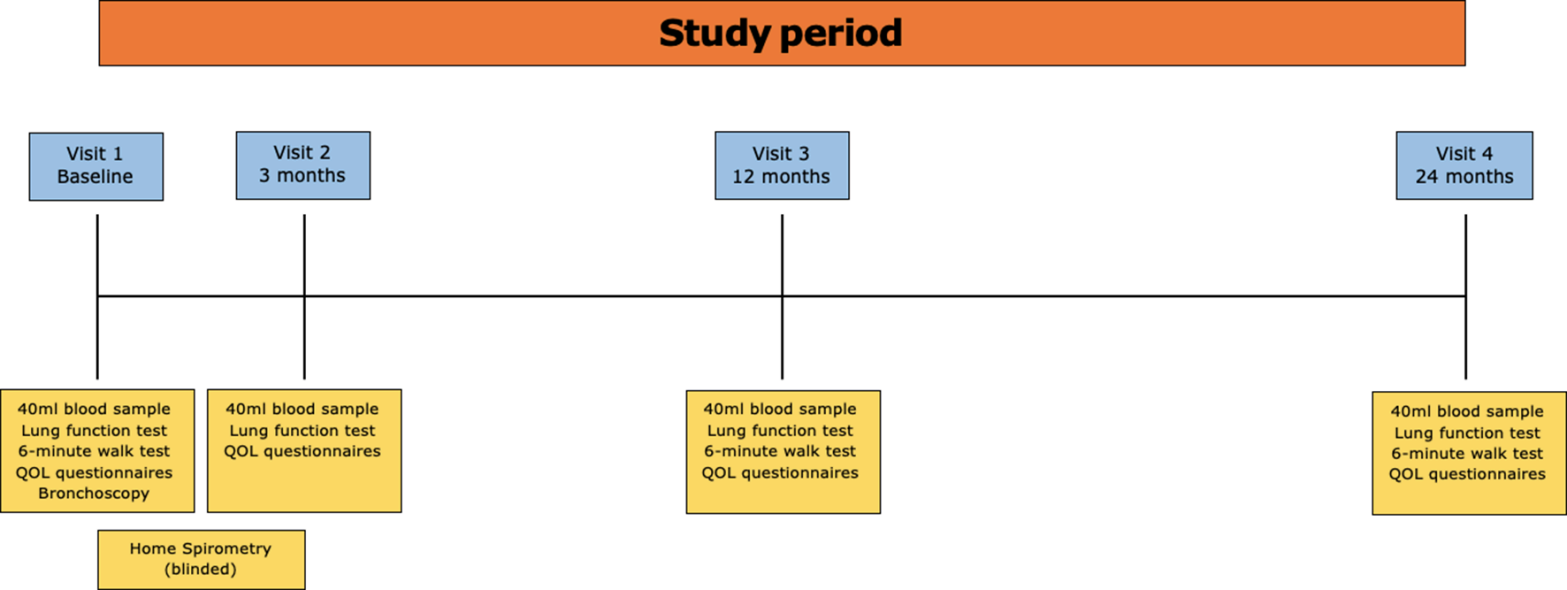
Legend – participant flow through the study.

Further visits at 3 months, 12 months and 24 months will include further 40 mL blood sampling, QoL and full lung function analysis. At 12 months and 24 months a 6 min walk test will be repeated (figure 1).

The majority of initial sample processing will be performed at the participant’s local NHS hospital trust via the Clinical Research Network. Participation in the study will be for 2 years. Other than initial assessment and follow-up visits at 3 months, 12 months and 24 months, there will be no further assessments. Only participants recruited at Nottingham University Hospitals NHS Trust will be offered a study bronchoscopy. It is not anticipated that any data will be obtained before completion of study that would lead to discontinuation of the study. For individual participants, discontinuation will be decided on an individual basis. The end of study will be defined as the last patient to complete the 2 years of follow-up.

### Specimen processing and analysis

All human tissue (blood, lavage, biopsy, etc.) samples will be processed and stored in accordance with the Human Tissue Act (2004). All biological samples will be processed within 2 hours of being obtained according to local standard operating procedures and stored in 500 µl aliquots at −80°C until analysis. Sample analysis will take place either at the University of Nottingham or in the laboratories of third party national, or international, academic partners or contract research organisations following appropriate tissue transfer agreements.

The primary analysis will include measurement of epithelial biomarkers [matrix metalloproteinase-7 (MMP-7), cancer antigen 125 (CA-125), carbohydrate antigen 19-9 (CA19-9), surfactant protein D (SP-D)]) and markers of matrix turnover [C-reactive protein degraded by MMP-1/8 (CRPM), collagen 3 degraded by MMP-9 (C3M) and collagen 6 degraded by MMP-2/9 (C6M)) as well as genotyping for mucin 5B (*MUC5B)*, desmoplakin (*DSP)* and A-kinase anchoring protein 13 (*AKAP13)*. Exploratory analysis will include whole genome sequencing, RNA sequencing, proteomic and metabolomic analysis to identify novel biomarkers that predict fibrotic disease behaviour. Biopsy material will be used to culture cellular components, frozen for extraction of protein, RNA and DNA or formalin-fixed paraffin embedded.

Any additional samples will be archived for future genetic and biomarker studies in the University of Nottingham premises at the Nottingham City Hospital. The Human Tissues Authority license number is 12 265. Where participants do not agree to the future use of the samples they will be destroyed in accordance with the Human Tissue Act, 2004.

#### Details of spirometry

A secondary objective of this study is to determine whether change in daily home (domiciliary) handheld spirometry values over 3 months can predict disease progression and overall survival.

Participants wishing to take part in home spirometry will be provided with a portable handheld spirometer (MIR Spirobank Smart) linked to an electronic health journal (patientMpower smartphone application) on enrolment. Participants will be trained to undertake daily spirometry readings (one forced expiratory manoeuvre/day; seated) for the first 3 months of the study period. All spirometry readings will be blinded to participants and automatically uploaded to patientMpower via the smartphone application, ensuring full encryption throughout. Participants will therefore need to possess, and be confident in using, a smartphone device and have an email address. The spirometry data will then be transferred to the University of Nottingham for further analysis. Participants will be able to continue to use the spirometer and patientMpower application with open display of FVC readings after the initial 3-month observation period if they wish.

#### Statistics

The primary end point of disease progression (10% relative FVC decline or death within 12 months) will be used dichotomously across all subgroups collectively analysed together, with the exception of IPF controls. Association of baseline biomarker levels with dichotomous disease progression and overall survival will be analysed using binomial family of generalised linear models, while Gaussian family or otherwise appropriate models will be used to assess associations with continuous secondary end points. The Benjamini-Hochberg procedure will be applied to account for multiplicity as appropriate. Repeated measures mixed models will be used to assess associations over time, which includes time points within one model and circumvents correction for multiple testing. Fixed factors will include baseline demographic information. Where putative biomarkers are identified, end point data will be used to compare biomarker levels by time-to-event through proportional hazard models; comparable ability to predict end points will be assessed through sensitivity and specificity analyses (receiver operator characteristic curves).

To identify transcriptomic biomarkers, RNA-seq libraries will be prepared and entered into a workflow for read count normalisation enabling quantification of transcript expression; normalisation ensures length and abundance of cDNA reads are corrected according to other genes (reads per kilobase per million) and further library scaling can occur.^25 26^ Libraries, aligned to an appropriate reference genome, will enable detection of differential gene expression and SNPs/variants according to primary end point and secondary measures of disease severity. Transcript-discovery artefacts, transcripts that remain below detectable levels of change across compared samples, as well as any with zero mapped reads will be excluded. R statistical packages will be used for these bioinformatics workflows.

Further statistical analyses will be performed to determine associations between identified biomarkers and exploratory secondary objectives. Statistical approaches include, but are not limited to, correlation and analyses of variance between biomarker levels and patient-reported QoL outcomes or disease exacerbations; machine learning strategies on home spirometry to detect decline in lung function, and subsequent comparison of sensitivity with traditional spirometry measures at predicting end points. Factor analysis approaches will be used to reduce dimensions of data sets and assess clustering; generalised linear models will be used to test associations.

**Figure 2:**
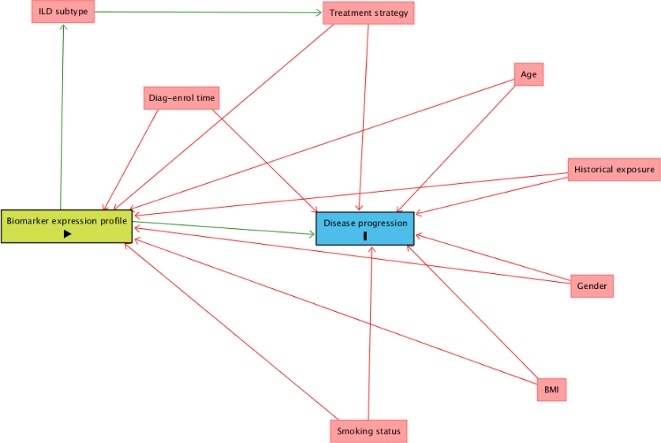
Directed acyclic graph (DAG) illustrates proposed model of causal inference and minimal sufficient adjustment sets (confounders; red) for estimating the direct effect of biomarker expression profiles (exposure; green) on disease progression (outcome; blue). Image generated using DAGitty.net environment. QoL, quality of life. BMI, body mass index; ILD, interstitial lung disease.

Confounding factors are particularly important in studies that hope to offer causal inference of biomarkers on clinical outcomes,^27^ and corrections to adjust for these will be applied in the analysis as proposed by the directed acyclic graph (figure 2).^28^ Fixed factors in mixed models will include baseline demographic information: age, gender, body mass index and smoking status. Exposure history in patients with chronic HP will be collected as will time from diagnosis to enrolment across all subgroups and adjusted for as a potential confounder. Some participants may be on background therapies such as immunosuppression and corticosteroids. While these therapies are likely to be confounders, it is possible they may decrease or increase progression of fibrosis based on the PANTHER (Prednisone, Azathioprine, and *N*-Acetylcysteine: A Study That Evaluates Response in Idiopathic Pulmonary Fibrosis) Study.^29^ Therefore, models will include treatment strategy as part of the minimal set of adjustment for estimating direct effect on end point. The objectives of the study do not extend to assessing treatment effect. Further unknown confounders will be identified from the spirometry smartphone application that will collect various data such as air quality index to inform limitations and future study.

### Sample size justification

A power calculation has been conducted using prior data obtained at 3 months in the PROFILE (Prospective Observation of Fibrosis in the Lung Clinical Endpoints) study of patients with IPF.^8^ The study demonstrated that 90 stable and 90 progressive individuals were sufficient to detect a twofold change in MMP7 with >90% power and 5% type I error rate. MMP7 was selected for power calculation as the most conservative of the biomarkers identified in the PROFILE Study, with the lowest threshold for biomarker change and least statistical precision, thus powering on MMP7 ensures that other biomarkers will be analysed with adequate power. The calculation demonstrates that a sample of 100 participants with progressive fibrosis and 100 with stable fibrosis will be large enough to detect dynamic differences in biomarkers over 3 months with 80% power and a type I error rate of 5% (α=0.05). Therefore 200 participants with ILD will be recruited assuming 50% disease progression. Fifty positive IPF controls will be recruited to take part in the study to benchmark progressive fibrotic lung disease but will not be included in the final analysis.

The study is also appropriately powered for genetic risk scores. An individual SNP with 25% minor allele frequency would have >70% power to detect an OR of 1.8 for stable versus progressive disease. The power for a risk score comprising multiple variants is expected to be greater. This assumes an additive genetic model, p<0.05, in 100 stable versus 100 progressive patients whereby with each additional marker added to the model the power is actually increased rather than reduced. The markers used will all be defined a priori based on data obtained from the PROFILE Study^7 8^ but are likely to include MUC5B, DSP, AKAP13, SP-D, CA-125, CA19-9, MMP-7, CRPM and C6M.

Following the recruitment of 100 participants who complete 1 year in the study, an interim sample size re-estimation by an independent data monitoring committee will be conducted to determine the progression status between blinded subgroups. If the blinded progression status is approximately 50%, recruitment will continue as described. If however, any subgroups show a relatively stable phenotype they will be excluded from subsequent recruitment, after being unblinded to the data monitoring committee. To attain an adequately powered sample size with 50% progressive fibrosis, further recruitment will be enriched with participants from the progressive phenotypical subgroups relative to rate of progression in interim analysis. Those recruited from subsequently excluded subgroups will be removed from the primary analysis, as they will be sources of inconsistency. However, if all subgroups progress at a substantially lower rate than expected and conditional power calculations at interim analysis suggest futility then all subgroups will be included in an analysis that demonstrates the null hypothesis (fibrotic lung disease progress at a rate specific to aetiology) could not be rejected. The study is not statistically powered to detect differences between ILD subgroups, although exploratory analyses will be carried out to inform future studies and support replication studies.

### Patient and public involvement

The Action for Pulmonary Fibrosis (APF)^30^ charity have been consulted during the design of the study and will sit on the steering committee as patient representatives, which will inform study conduct and recruitment. All patient information material has been reviewed by patient representatives. Study findings will be communicated to participants, and the APF will also support the dissemination of the study’s finding to patients with pulmonary fibrosis and their families.

## Ethics and dissemination

### Monitoring

Monitoring of study data will include confirmation of informed consent; source data verification; data storage and data transfer procedures; local quality control checks and procedures, backup and disaster recovery of any local databases and validation of data manipulation. Entries on case report forms (CRFs) will be verified by inspection against the source data. A sample of CRFs (10% or as per the study risk assessment) will be checked on a regular basis for verification of all entries made. In addition, the subsequent capture of the data on the study database will be checked. Where corrections are required, these will carry a full audit trail and justification.

The study coordinator, or where required, a nominated designee of the sponsor, shall carry out monitoring of study data as an ongoing activity Trial data and evidence of monitoring and systems audit will be made available for inspection by the research ethics committee as required.

### Safety reporting

No significant safety concerns are anticipated in relation to any measurements carried out as part of this trial. For patients undertaking bronchoscopy, the possible risks are the same as described in the hospital information sheet given prior to the procedure. All adverse events will be recorded and closely monitored until resolution, stabilisation or until it has been that the study intervention is not the cause. The chief investigator shall be informed immediately of any serious adverse events and shall determine seriousness and causality in conjunction with any treating medical practitioners.

### Trial monitoring and oversight

The trial will be overseen by the INJUSTIS steering group consisting of the chief investigator, centre manager, research officer, research fellow, statistician, patient representatives (APF) and an independent chairperson. This committee will meet every 4 months.

Interim analysis will be undertaken by an independent data monitoring committee that will comprise two clinicians with expertise in clinical trials in ILD and a statistician not directly involved in this study.

### Dissemination

All data will be anonymised and grouped for presentation and publication. The results from this study will be publicised at regional and national conferences as well as being submitted for publication in open access peer-reviewed journals in accordance with UK Research Council policies. No participants will be identified in any publications that arise from this research.

## Conclusion

The INJUSTIS is a prospective longitudinal study of non-idiopathic fibrotic ILD, that will identify biomarkers of progression of fibrotic lung disease regardless of aetiology should such biomarkers exist. However, this study is not powered to detect differences between fibrotic lung diseases of specific aetiologies, although it may provide insights into specific fibrotic lung diseases for further investigation.
